# Impact of n,γ-irradiation on organic complexes of rare earth metals

**DOI:** 10.1038/s41598-019-49962-9

**Published:** 2019-09-16

**Authors:** Tatyana V. Balashova, Sergey V. Obolensky, Alexey N. Trufanov, Mikhail N. Ivin, Vasily A. Ilichev, Andrey A. Kukinov, Eugeny V. Baranov, Georgy K. Fukin, Mikhail N. Bochkarev

**Affiliations:** 1 G. A. Razuvaev Institute of Organometallic Chemistry of Russian Academy of Sciences, Tropinina 49, 603950 Nizhny Novgorod, Russian Federation; 2 Nizhny Novgorod State University, Gagarina avenue 23/2, 603950 Nizhny Novgorod, Russian Federation; 3 Branch of RFYaTs-VNIIEF «Yu.E. Sedakov FNCP NIIIS», Tropinina 47, 603950 Nizhny Novgorod, Russian Federation

**Keywords:** Organometallic chemistry, Experimental nuclear physics

## Abstract

The complexes of La, Ce, Nd, Sm, Eu, Tb and Yb with benzoxazolyl-phenolate, benzothiazolyl-phenolate, benzoxazolyl-naphtholate, benzothiazolyl-naphtholate and 4,4,4-trifluoro-1-(2-thienyl)-1,3-butanedione ligands were treated with n,γ-irradiation upon a sustained (45 h, absorbed dose of 120 krad, flux of neutrons 5·10^13^ n/cm^2^) and a pulse mode (3 ms, absorbed dose of 130 krad, flux of neutrons 3.6·10^13^ n/cm^2^). It was found that main characteristics of the compounds (shape of substance, color, IR absorption and photoluminescent spectra) have not changed. With an example of cerium complex [Ce(OON)_3_]_2_ it was revealed that the molecular structure of compounds after strong pulse irradiation also does not changed. However, computer simulations of neutron exposure on the same complexes showed significant shift of metal atoms and ligands. Possible reasons for the detected discrepancy between experimental and calculated data are discussed.

## Introduction

Many organic complexes of lanthanides, due to the peculiarities of the electronic structure of the metal atom, possess luminescent and photovoltaic properties, which determine their use in modern optoelectronic devices and in biomedicine^1^. All methods and technologies where rare earth metal complexes are used assume their work under “normal” conditions, i.e. in the absence of specific forms of exposure. However, over the past century, in the sphere of human activity, new areas have emerged and are rapidly expanding, where a new component — ionizing irradiation (InI) — is being added to ordinary conditions. Such areas include space, active zones of nuclear power plants, concentrating and processing radioactive materials enterprises, radio- therapy and diagnostics, territories polluted with nuclides as a result of accidents. Unfortunately, it is impossible to exclude the existing probability of contamination of the area by nuclides due to the explosion of nuclear warheads. Currently, in devices designed to operate under InI conditions, only inorganic semiconductor materials are used, although studies have shown their relatively low radiation resistance^2–5^. There are as well two papers on the radiation resistance of organic semiconductors in which the polymer/fullerene systems are considered as promising for designing solar cells operating under radiation conditions^6,7^. Metal-organic substances, as an alternative to inorganic analogues, have not been previously considered, despite the fact that in many respects (flexibility, weight, tunability of various characteristics, cost, processability) the latter are significantly inferior to organic derivatives. The main reason for this situation was, apparently, the a priori prevailing opinion about the low radiation resistance of metal-organic compounds. The conclusion was based on a comparison of the thermal, chemical, and photolytic stability of both types of substances. As far as we know, no special studies have been carried out on the radiation resistance of organic complexes. However, recently published data on the radioluminescent properties of aryl carboxylates of non-transition^8,9^, d-transition metals^10^ and lanthanides^11^ indirectly indicate that substances of this class may have sufficiently high radiation resistance.

In this paper, we report on the study of the effects of n,γ-radiation on lanthanide organic complexes in order to determine the possibility of their use in devices operating under conditions of enhanced radiation. As objects, we selected complexes with substituted phenolate and naphtholate ligands, the main physico-chemical characteristics and molecular structure of which were described by us earlier^12–14^. A modified well-known europium 2-thenoyltrifluoroacetonate Eu(TTA)_3_(DME)_2_, which has a different type of ligands and has good luminescent properties, was also used.

## Results and Discussions

All the complexes chosen for the study are quite stable in an inert atmosphere and in vacuum but hydrolyze slowly in air except the air-stable Eu compound. Upon heating they decompose above 180°С. The compounds [Nd(NpSON)_3_]_2_, [Sm(OON)_3_]_2_, Eu(TTA)_3_(DME)_2_, [Tb(OON)_3_]_2_ and [Yb(NpSON)_3_]_2_ when excited by UV light generate intense metal-centered emission. Cerium complex shows only the ligand luminescence. For some compounds, including [Ce(OON)_3_]_2_, the molecular structure has been determined by X-ray diffraction method^12^. The listed properties, which are convenient for the identification of compounds, stipulated our choice of these substances as models for studying the radiation stability of metal-organic complexes.

**Figure 1:**
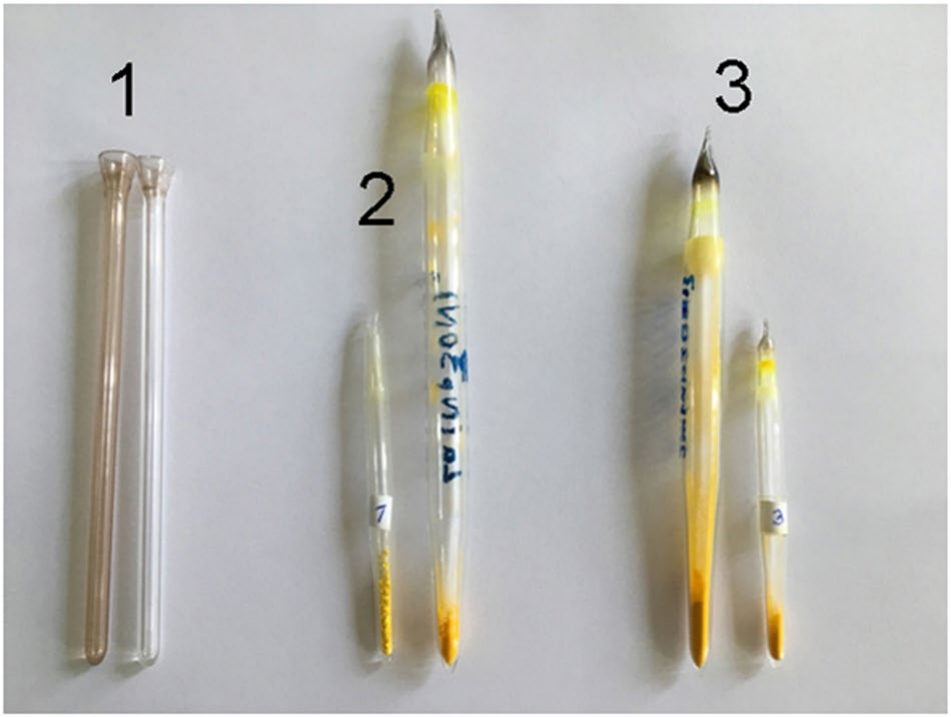
Photo of the samples of the compounds [La(NpSON)_3_]_2_ (2), [Sm(NpSON)_3_]_2_ (3) and the glass tubes (1) before (right sample) and after (left sample) irradiation.

The samples were processed by radiation generated upon the decay of ^235^U nuclei. The full spectrum of radiation was used including α, ß, γ components and neutrons. However, since α and ß-components were filtered almost completely upon passage through the walls of the glass tubes, the material was exposed only by neutrons and γ-radiation. The study included three stages: the first two were carried out in a sustained mode (45 h), the third stage was pulse irradiation (3 ms). The dose of absorbed radiation, with the neutron flux of 6·10^12^, 5·10^13^ and 3.6·10^13^ n/cm^2^, was 12, 120 and 130 krad, respectively. After irradiation, the color and appearance of the samples remained almost unchanged (Fig. 1). Since the color of the glass tube after pulse irradiation changed (control sample 1), the complexes were overloaded into a new tube before photographing.

**Figure 2:**
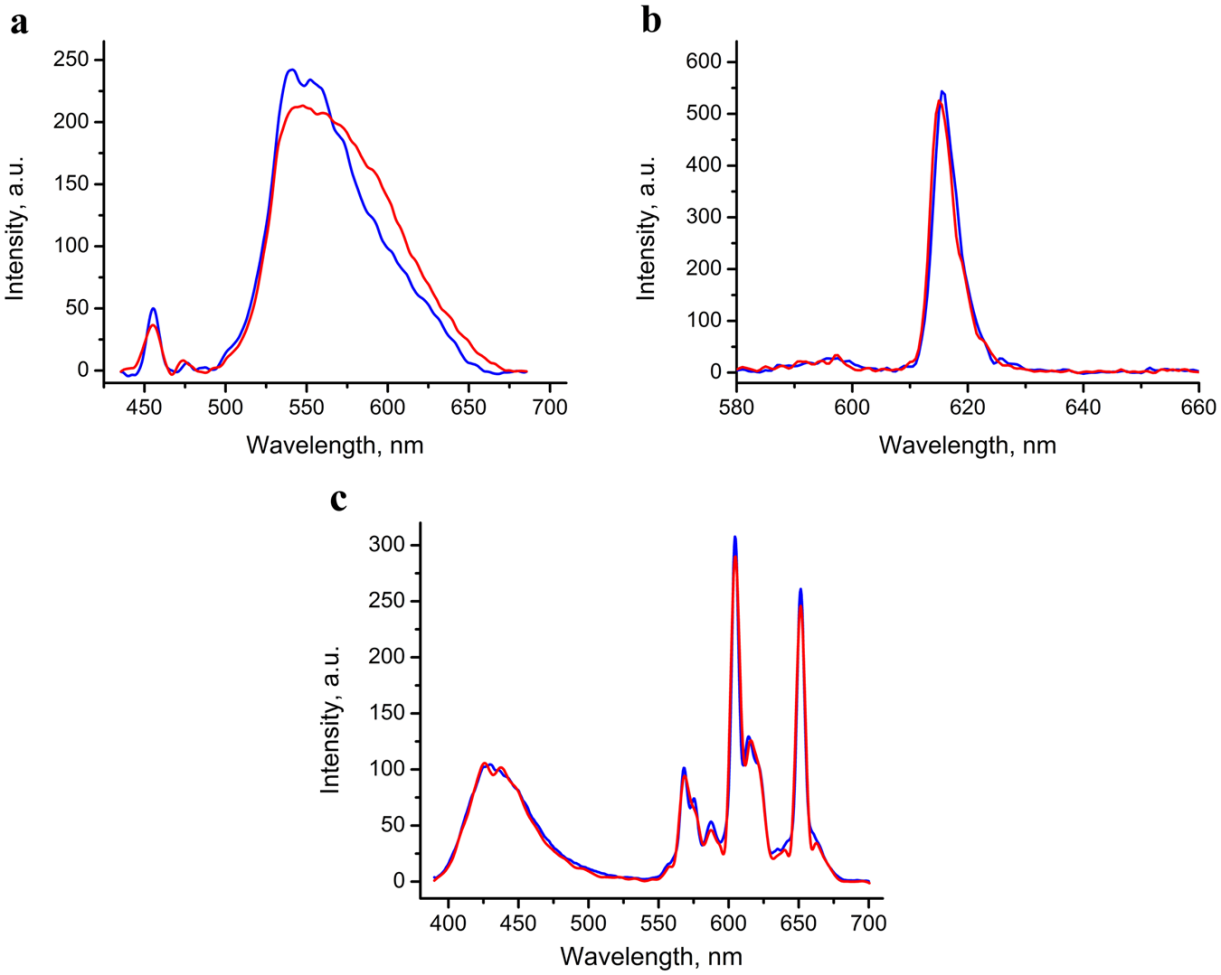
Photoluminescent spectra of irradiated (red) and non-irradiated (blue) solid complexes [La(NpSON)_3_]_2_ (**a**) (excitation 395 nm), Eu(TTA)_3_(DME)_2_ (**b**) (excitation 370 nm) and THF solution of [Sm(OON)_3_]_2_ (**c**) (excitation 370 nm).

Comparison of the IR spectra of the irradiated samples of the [La(NpSON)_3_]_2_, [Sm(OON)_3_]_2_, and Eu(TTA)_3_(DME)_2_ complexes with the spectra of the corresponding samples that were not processed did not reveal differences (Fig. S1). The set and intensity of the bands in the photoluminescence spectra of irradiated and non-irradiated solid samples of the same compounds also displayed no difference (Fig. 2).

The open-circuit voltage (0.3 V), short current (22 μA/cm^2^) and fill factor (15%) of the photovoltaic cell based on the irradiated sample of the ytterbium complex [Yb(NpSON)_3_]_2_ coincided with the corresponding characteristics of the cell containing the non-irradiated compound. It should be noted that the semiconductor properties of such widely used inorganic materials as Si, SiN, GaAs are changed significantly and irreversibly already when irradiated with an energy of 200 keV and an absorbed dose of 905 Gy (90.5 krad)^2^.

**Figure 3:**
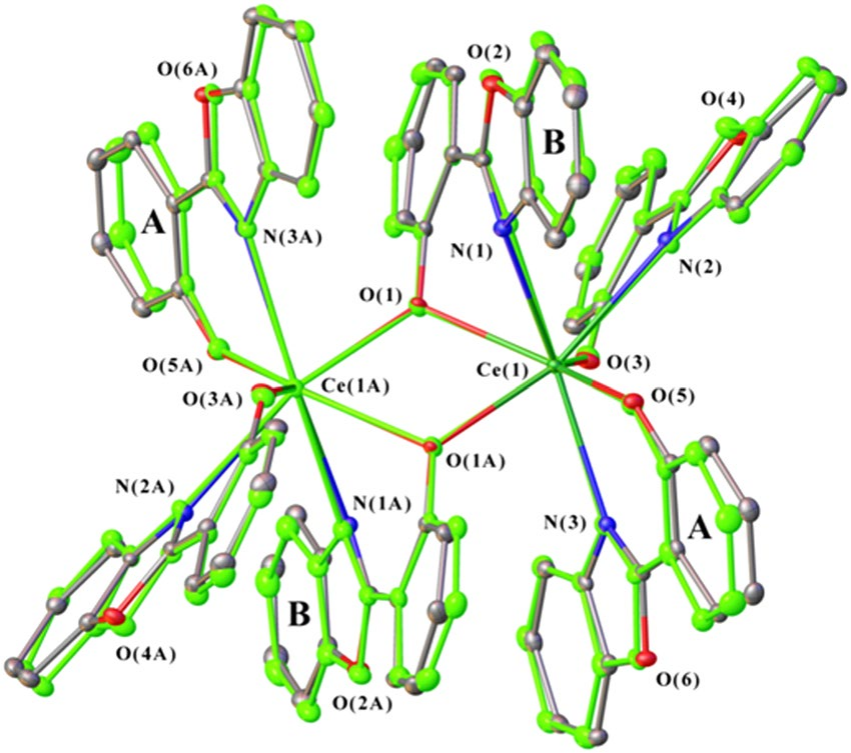
Molecular structure of the irradiated (grey) and non-irradiated (green) [Ce(OON)_3_]_2_ complex. Thermal ellipsoids are drawn at 30% level probability. Hydrogen atoms are omitted for clarity.

The crystals of the [Ce(OON)_3_]_2_ complex even after intense pulse irradiation retained their shape, which made it possible to use X-ray diffraction analysis to determine their molecular structure. Note that the study of the degradation of organic and metal-organic compounds under the action of InI at the molecular level has not previously been carried out. The analysis data showed that the structure of the treated complex is very slightly different from the structure of the compound which was not irradiated and the complex studied six years ago^12^. Differences are manifested in a negligible change in the position of the ligands (Tables S1 and S2). The greatest displacement of 0.482 Å is observed for C atoms of phenyl groups of terminal ligands. In Fig. 3 the molecular structure of irradiated and non-irradiated [Ce(OON)_3_]_2_ complex are shown. For convenience of comparison, both structures are superimposed on one another in such a way that the metal atoms and the bridging oxygen atoms are superposed.

The data obtained gave grounds to suggest that n,γ-irradiation caused some changes in the molecular structure of the studied complex. To test the assumption we compared the unit cell parameters of the irradiated compound with those of the earlier obtained related complexes [Pr(OON)_3_]_2_^15^, [Eu(OON)_3_]_2_ and [Gd(OON)_3_]_2_^12^, which were not exposed to InI, and found that these parameters exactly correspond to the values of irradiated [Ce(OON)_3_]_2_. This prompted us to repeat the synthesis of the Ce complex and examine again its structure. The cell parameters of the newly synthesized compound coincided with the parameters of the irradiated sample. Thus, the data obtained showed that (i) InI has not destroyed the crystal and molecular structure of the treated complex and (ii) the synthesis of organo-lanthanide complexes can give products with the same arrangement but with a different structure, even under the same preparation conditions.

The fact that the crystal lattice of the metal complexes is preserved after irradiation with high intensity is unexpected, especially since computer simulation of the effect of fast neutrons on the same compound gives the opposite result. Calculations show that the effect of neutrons with energy of 500 keV on cerium atoms, as well as on other atoms, will cause their displacement by more than 100 nm from the initial position, i.e. should lead to the destruction of the substance (Fig. S2). Found contradiction experimental and calculated data is probably explained by the relatively low density of the applied neutron radiation: a flux was no more than 5·10^13^ n/cm^2^. Since the sample contained about 1·10^19^ Ce atoms, it is quite clear that even under the assumption that each neutron reaches the target, only an insignificant part of the metal and other atoms undergoes an attack and this atoms are unable to exert a noticeable influence on the overall structure of the complex and consequently on the optoelectronic properties of the substance.

All the used compounds after irradiation become radioactive due to the capture of thermal neutrons by atomic nuclei in the complexes. Magnitudes of the induced radioactivity in three days after treatment (4.56, 6.29 and 4.52 μZ for La(NpSON)_3_, Sm(NpSON)_3_ and Eu(NpSON)_3_, respectively) indicate the formation of new isotopes of rare earth metals since radioactive isotopes of other elements in the complexes (C, O, S and N) have very short half-life and by the time of measurement the complexes contain only stable isotope of these element. As these data indicate nuclear reactions in metal-organic compounds, the question arises whether these reactions cause the destruction of the molecule of the complex or its structure remains unchanged? As it was showed by many previous studies, the nuclear reactions on the organometallic complexes as a rule lead to splitting off of the organic ligands and in some cases to a deep transformation of the latter^16,17^. This way is used for a preparation of new radioactive organometallic complexes^18–20^ in particular the complexes for neutron capture therapy^21,22^. In all these nuclear synthesis with organometallic compounds very few atoms (10^−10%^)^16^ undergo transformation. In our case, as shown by X-ray diffraction studies of a cerium compound, the most of molecules in the lanthanide coordination complexes are not destroyed upon neutron bombardment of the material. This, obviously, explains their relatively high radiation stability and the preservation of the optoelectronic properties under the action of n,γ-irradiation.

## Methods

Complexes of La, Ce, Nd, Sm, Eu, Tb and Yb with 2-(2-benzoxazol-2-yl)phenolate (OON)^12^ and 3-(2- benzoxazol-2-yl)-2-naphtholate (NpSON)^13^ ligands were synthesized as described earlier. Europium 2-thenoyltrifluoroacetonate Eu(TTA)_3_(DME)_2_ was synthesized under anaerobic conditions by the reaction of Eu[N(SiMe_3_)_2_]_3_ (67 mg, 0.106 mmol) with tris(4,4,4-trifluoro-1-(2-thienyl)-1,3-butanedione) (H(TTA)) (70 mg, 0.315 mmol) in DME (10 ml) at room temperature (10 min.) and isolated in the form of a light yellow powder. Yield 81 mg (76%). Anal. Calcd. (%) for C_32_H_35_EuF_9_O_10_S_3_ (998.76) C, 38.48; H, 3.53; Eu, 15.22; S, 9.63. Found (%) C, 38.46; H, 3.57; Eu, 15.26; S, 9.59. IR (KBr, ν, cm^−1^): 1623 (s), 1600 (s), 1577 (s), 1540 (s), 1511 (s), 1413 (s), 1395 (s), 1306 (s), 1256 (m), 1233 (m), 1187 (m), 1139 (s), 1056 (m), 1030 (m), 936 (m), 865 (m), 791 (s), 770 (w), 710 (m), 685 (m), 643 (s), 583 (s). The C, H, N elemental analyses were performed by the Microanalytical laboratory of IOMC on Euro EA 3000 Elemental Analyser. The lanthanides content was analyzed by complexometric titration. IR spectra were obtained on a FTIR spectrometer FSM-1201 and recorded from 4000 to 400 cm^–1^ as a Nujol mull on KBr plates. Emission spectra were registered from 300 to 700 nm on a fluorescent spectrometer Perkin Elmer LS-55. The prototypes of photovoltaic cells were designed and tested as described elsewhere^23^.

Samples of the complexes (100–150 mg) in the form of a fine crystalline powder were placed in glass tubes with a diameter of 2.5 mm and a wall thickness of 1 mm. The tubes were evacuated, sealed and treated with n,γ-irradiation for 45 h at a sustained mode. The absorbed dose of radiation was 12 krad with a flux of neutrons of 6 ·10^12^ n/cm^2^. The same samples were repeatedly treated for 45 hours with more intense radiation (absorbed dose of 120 krad with a flux of 5·10^13^ n/cm^2^). Samples of the complexes [La(NpSON)_3_]_2_, [Nd(NpSON)_3_]_2_, [Sm(NpSON)_3_]_2_, [Eu(NpSON)_3_]_2_ in the form of fine-crystalline powders and the cerium complex [Ce(OON)_3_]_2_ in the form of crystals, prepared as in previous experiments^12^, were irradiated with an pulse mode (duration of 3 ms). Absorbed dose was 130 krad at a flux of neutrons of 3.6·10^13^ n/cm^2^. After each irradiation, the IR spectrum was recorded for all compounds. For the compounds [Nd(NpSON)_3_]_2_, [Sm(OON)_3_]_2_ and Eu(TTA)_3_(DME)_2_ the photoluminescence spectra of solid samples and THF solutions were recorded. For the sample of the complex [Yb(NpSON)_3_]_2_, the photovoltaic characteristics were determined in the planar photovoltaic cell of configuration ITO/complex (25 nm)/C_60_ (40 nm)/Bphen (8 nm)/Al (Bphen – 4,7-diphenyl-1,10-phenanthroline) which was fabricated by vacuum evaporation method as described earlier^24^.

In the case of the cerium complex [Ce(OON)_3_]_2_ after irradiation, its molecular structure was studied by X-ray diffraction method using a Bruker D8 Quest diffractometer (MoKα radiation, *ω*-and *φ*-scan technique, λ = 0.71073 Å). The intensity data were integrated by *SAINT* program^25^. *SADABS*^26^ was used to perform area-detector scaling and absorption corrections. The structure was solved by dual-space^27^ method and refined on F^2^ using all reflections with the SHELXTL package^28,29^. All non-hydrogen atoms were refined anisotropically. H atoms were placed in calculated positions and refined in the “riding model”. The details of crystallographic, collection and refinement data for [Ce(OON)_3_]_2_ are presented in Table S1 and S2. CCDC-1877840 contains the supplementary crystallographic data for this paper. These data can be obtained free of charge from the Cambridge Crystallographic Data Centre via www.ccdc.cam.ac.uk/data_request/cif.

A mathematical model of the destruction of the cerium complex [Ce(OON)_3_]_2_ under the neutron irradiation was carried out by the Monte Carlo method using the SRIM-2003 software package^30^. The calculation of the cross sections for the interaction of neutrons with atoms of the complexes was performed for average neutron energy of 2 MeV. Additional calculations were made for energy of 100 keV and 10 MeV. In the course of calculations, the neutron radiation energy was varied, and a trial calculation of the effect of gamma rays was also carried out. A comparison of the processes of defect formation in the studied materials was made for light and heavy atoms in a substance. The trajectory of each recoil atom, which received energy upon the impact of a neutron, began with the input of its position, direction of motion, and energy. Over the free path length of the recoil atoms, their energy decreased by the amount of electronic losses for ionization of the material, and then, after a collision, by so-called nuclear, or elastic energy losses, i.e. on the energy transferred to the target atom in a collision. The energy transferred by neutrons to recoil atoms and responsible for radiation violations was calculated by the formula (1)^30^

1 $${T}_{a}=\frac{4{M}_{n}{M}_{A}}{{({M}_{n}+{M}_{A})}^{2}}{T}_{n}{\sin }^{2}(\frac{{\rm{\theta }}}{2})=\frac{4A}{{(1+{A}^{2})}^{2}}{T}_{n}$$

where М_n_, М_А_ is the mass of a neutron and an atom; T_n_ is the kinetic energy of a bombarding neuron; θ is the recoil angle between the direction of motion of the neutron before and after the collision, A is the atomic weight.

The angle of scattering in the centre of mass system was defined as in the formula (2):

2 $$\cos \,{\rm{\theta }}=\frac{P+p+\delta }{p+{r}_{0}}{T}_{n}$$

The selection of target atoms was made using random numbers. It was assumed that the probability of a collision with an atom of each type is proportional to its stoichiometric coefficient determined by the brutto-formula. The difference between light and heavy atoms in the target was the magnitude of the interaction potential.

In the model of nonlocal energy loss, electron loss was determined as the multiplying of the braking function and the length of free path of the recoil atom. We used the Bragg rule, which states that the contribution to the stopping power of each type of target atoms is proportional to their atomic fraction.

## Conclusions

Thus, with the example of organo-lanthanide complexes, the radiation resistance of molecular metal-organic compounds was studied for the first time. It was found that the stability of the studied compounds with respect to the effects of n- and γ-radiation exceeds the stability of inorganic semiconductor materials. Study of the radiation resistance of organic compounds of lanthanides at the molecular level with the example of the cerium complex showed that the bombardment of the tested substances with fast neutrons with energy of ∼1 MeV and flax 5·10^13^ n/cm^2^ does not cause changes in the structure of most molecules, so the photophysical characteristics of the material are preserved. The found high radiation resistance of organo-lanthanide complexes opens up the possibility of their use as functional materials in the design of devices and apparatus designed to work under conditions of enhanced radiation.

## Supplementary information

Supplementary Info
