# Supplementary material for: Impact of n,γ-irradiation on organic complexes of rare earth metals
Source: Sci Rep. 2019 Sep 16;9:13314. doi: 10.1038/s41598-019-49962-9 (PMC6746760; doi:10.1038/s41598-019-49962-9)
Supplement: Supplementary file 1 — Supplementary Info [file 41598_2019_49962_MOESM1_ESM.docx]

Supplementary materials

**Impact of n,γ-irradiation on organic complexes of rare earth metals**

Tatyana V. Balashova,^a^ Segey V. Obolensky,^b^ Alexey N. Trufanov,^c^ Mikhail N. Ivin,^b,c^ Vasily A. Ilichev,^a^ Andrey A. Kukinov,^a,b^ Eugeny V. Baranov,^a^ Georgy K. Fukin^a^ and Mikhail N. Bochkarev^a^

^a^ G. A. Razuvaev Institute of Organometallic Chemistry of Russian Academy of Sciences, Tropinina 49, 603950, Nizhny Novgorod, Russian Federation

^b^ Nizhny Novgorod State University, Gagarina avenue 23/2, 603950, Nizhny Novgorod, Russian Federation

^c^ Branch of RFYaTs-VNIIEF «Yu.E. Sedakov FNCP NIIIS», Tropinina 47, 603950, Nizhny Novgorod, Russian Federation


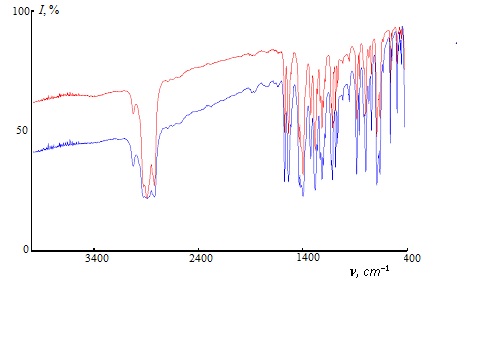
(a)


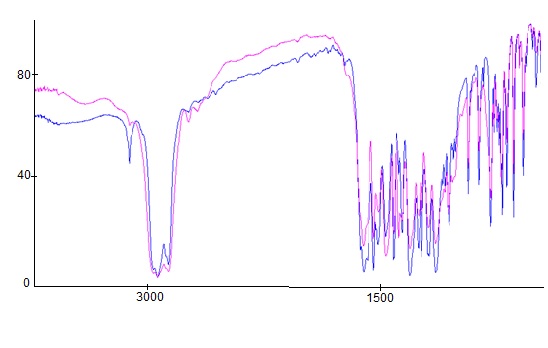
(b)


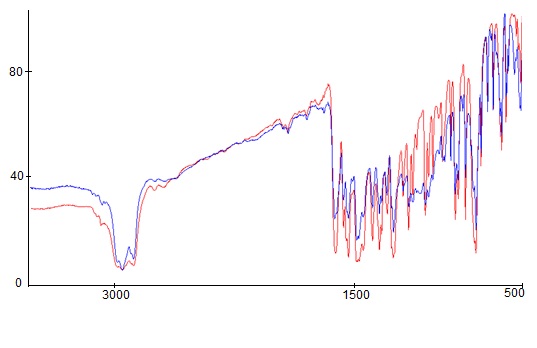
(c)

**Fig. S1**. IR spectra of [La(NpSON)_3_]_2_ (a), Eu(TTA)_3_(DME)_2_ (b) and [Sm(OON)_3_]_2_ (c) before (blue) and after (red) n,γ- exposure (τ 3 ms, neutron flux 3.6⋅10^13^ n/cm^2^, dose of absorbed radiation 130 krad).


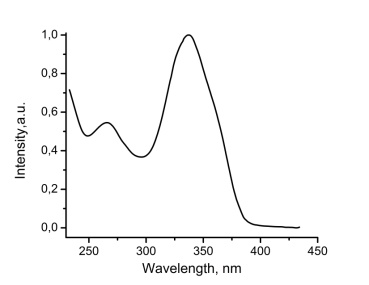

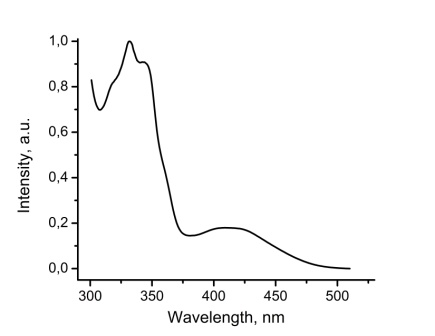

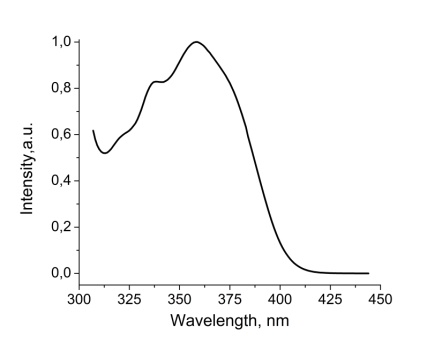


(a) (b) (c)

**Fig. S2**. Absorption spectra of Eu(TTA)_3_(DME)_2_ (a), [La(NpSON)_3_]_2_ (b) and [Sm(OON)_3_]_2_(c).


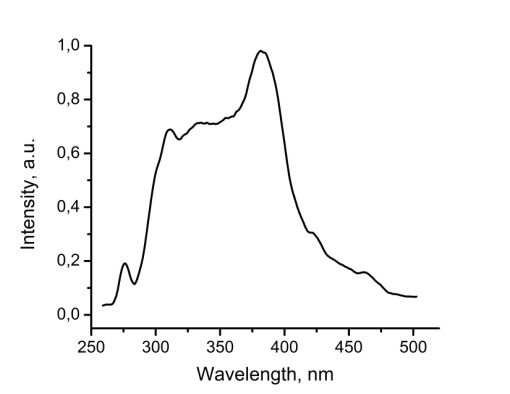

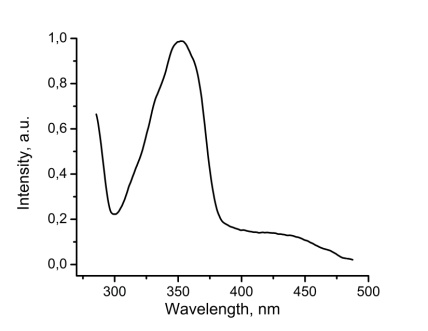

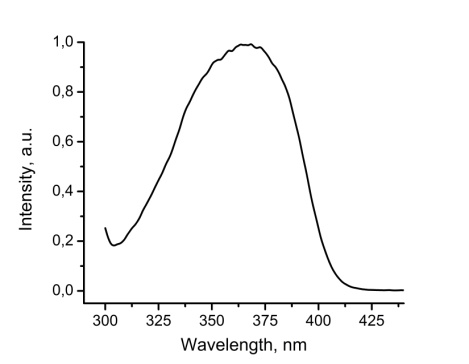


(a) (b) (c)

**Fig. S3**. Excitation spectra of Eu(TTA)_3_(DME)_2_ (a) (emission 617 nm), [La(NpSON)_3_]_2_ (b) (emission 550 nm) and [Sm(OON)_3_]_2_(c) (emission 605 nm).

**Table S1**. Crystallographic, collection and refinement data for irradiated sample of [Ce(OON)_3_]_2_.

| Empirical formula | C_78_H_48_Ce_2_N_6_O_12_ |
| --- | --- |
| Formula weight | 1541.46 |
| Crystal system | Triclinic |
| Space group | P-1 |
| *Т* [К] | 100(2) |
| *a* [Å] | 10.8572(3) |
| *b* [Å] | 12.0631(4) |
| c [Å] | 12.3703(3) |
| *α* [°] | 87.349(1) |
| *β* [°] | 76.344(1) |
| *γ* [°] | 78.290(1) |
| *V* [Å^3^] | 1541.59(8) |
| *Z* | 1 |
| *ρ* [г/см^3^] | 1.660 |
| *m* [мм^-1^] | 1.533 |
| *F*(000) | 770 |
| Crystal size [мм] | 0.246 × 0.240 × 0.143 |
| Range for data collection, *θ* [°] | 2.28 - 28.00 |
| Limiting indices | -14 ≤ *h* ≤ 14  -15 ≤ *k* ≤ 15  -16 ≤ *l* ≤ 16 |
| Reflections collected | 16690 |
| Unique reflections | 7431 [R_int._ = 0.0247] |
| Absorption correction  (max/min) | SADABS (0.7904 / 0.7130) |
| Data / Restraints / Parameters | 7431 / 0 / 442 |
| GOF (*F*^2^) | 1.051 |
| *R*_1_ / *wR*_2_ (*I* > 2*s*(*I*)) | 0.0228 / 0.0509 |
| *R*_1_ / *wR*_2_ (all data) | 0.0278 / 0.0521 |
| Largest diff. peak and hole [e/Å^3^] | 0.757 / -0.703 |

**Table S2**. Selected bond lengths (*d*) and angles (*ω*) of [Ce(OON)_3_]_2_ in unirradiated (**I**) irradiated **II** samples.

| **Bond** | *d*, Å  **I** | *d*, Å  **II** | Angle | *ω*, °  **I** | *ω*, °  **II** |
| --- | --- | --- | --- | --- | --- |
| Ce-O^br^ | 2.436(2)  2.437(2) | 2.4248(13),  2.4482(13) | O^br^-Ce-O^br^ | 67.57(6) | 68.51(5) |
| Ce-O^term^ | 2.285(2),  2.300(2) | 2.2889(14),  2.2953(15) | N^br^-Ce-O^br^ | 66.69(6) | 66.41(5) |
| Ce-N^br^ | 2.672(2) | 2.6688(16) | N^term^-Ce-O^term^ | 68.39(6),  70.03(6) | 68.40(5),  70.13(5) |
| Ce-N^term^ | 2.595(2),  2.641(2) | 2.5957(15),  2.6584(16) | Ce-O^br^-Ce | 112.43(6) | 111.49(5) |
| Ce…Ce | 4.0499(3) | 4.0279(2) |  |  |  |

br – a bridging ligand, term – a terminal ligand


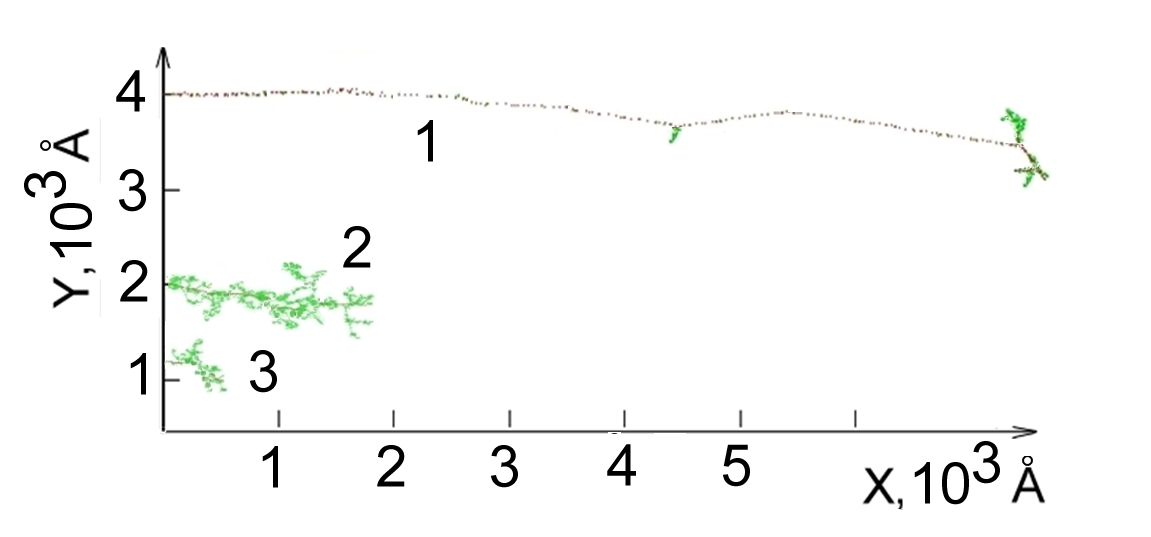


**Fig. S2**. Cascades of recoil atoms formed by primary atoms: 1 - O (443 keV), 2, 3 - Ce (443 and 56 keV, respectively) in [Ce(OON)_3_]_2_.

Each point of the image corresponds to the displaced atom. It is assumed that the neutron transmitted energy to the atoms at the advising starting point on the vertical axis, and their primary impulse is directed to the right. The primary recoil atom, when moving from “left to right”, forms a chain of displaced atoms along its trajectory. Secondary recoil atoms, which received significant energy from the primary, form subcascades of displaced atoms.
